# Health-related quality of life among working-age women with breast cancer in Croatia: a population-referenced cross-sectional study

**DOI:** 10.1186/s12905-026-04562-4

**Published:** 2026-05-26

**Authors:** Vid Duplančić, Ana Bobinac, Luka Vončina, Katarina Hraste, Ana Tečić Vuger, Robert Šeparović, Eduard Vrdoljak

**Affiliations:** 1 Department of Radiotherapy and Oncology, University Hospital Center Split, School of Medicine, University of Split, Split, Croatia; 2 Faculty of Economics and Business, University of Rijeka, Rijeka, Croatia; 3 Faculty of Health Studies, University of Rijeka, Rijeka, Croatia; 4 Division for Medical Oncology, University Hospital for Tumors, Sestre Milosrdnice University Hospital Center, Zagreb, Croatia

**Keywords:** Breast cancer, Health-related quality of life, EQ-5D-5L, EORTC QLQ-BR23, Population norms, Working-age women, Croatia, Survivorship care

## Abstract

**Background:**

Health-related quality of life (HRQoL) is a key outcome in breast cancer survivorship, yet population-referenced data for working-age women, a group facing distinct social and functional challenges, in Central and Eastern Europe remain scarce. We aimed to quantify HRQoL among Croatian working-age women with breast cancer and to benchmark patient-reported outcomes against national female population norms.

**Methods:**

We conducted a multicentre cross-sectional study in 2024 at two tertiary oncology centres in Croatia. Women aged 18–65 years attending outpatient breast cancer care completed the EQ-5D-5 L (dimensions, EQ-VAS and utility index) and the EORTC QLQ-BR23. EQ-5D-5 L utilities were calculated using the Slovenian value set and compared with age-stratified Croatian female population norms (2022). Group differences were analysed using appropriate non-parametric tests.

**Results:**

In total, 271 women participated, with 233 included in EQ-5D-5 L analyses. Mean EQ-5D-5 L utility was substantially lower among patients than in the general female population (0.76 vs. 0.91), accompanied by lower EQ-VAS scores (66.2 vs. 84.0). Problems were most frequently reported in pain/discomfort and anxiety/depression. HRQoL declined with age (utility 0.82 to 0.70; *p* = 0.03) and was significantly lower in women with metastatic disease compared with non-metastatic disease (0.70 vs. 0.77; *p* = 0.03). Disease-specific assessment revealed pronounced impairments in future perspective and sexual functioning/enjoyment, alongside notable symptom burden related to systemic therapy side effects and arm and breast symptoms.

**Conclusion:**

Working-age women with breast cancer in Croatia experience clinically meaningful reductions in HRQoL compared with national population norms, with higher burden among older women and those with metastatic disease. These findings highlight substantial unmet survivorship care needs and support the integration of routine patient-reported outcome monitoring into oncology follow-up, alongside targeted symptom management, psychosocial support, rehabilitation and sexual health care.

## Background

Breast cancer represents the most common malignancy among women worldwide and one of the leading causes of premature mortality and health-related quality of life (HRQoL) reductions [1, 2]. Beyond its clinical consequences, extensive evidence highlights the profound psychological and social impacts of breast cancer on patients, survivors, and their families [3–5]. These challenges are particularly pronounced among younger, working-age women, who often experience disruptions in family roles, psychosocial functioning and daily activities.

While numerous studies in Western Europe have examined the HRQoL and psychosocial burden of breast cancer, evidence from Central and Eastern Europe (CEE) remains limited, with regional expert statements and policy reports pointing to broader gaps in cancer research capacity, inequalities in care, and underrepresentation of patient-reported outcomes in oncology research and practice across the region [6–9]. Given substantial cross-regional differences in socioeconomic conditions, cultural norms and healthcare resources, findings from Western European settings may not adequately reflect the lived experiences of patients in CEE countries. This highlights the importance of generating context-specific HRQoL data to inform national survivorship strategies and support services. Moreover, country-specific HRQoL data and benchmarking against national population norms are important for interpreting patient-reported outcomes and informing survivorship services. We therefore assessed HRQoL in working-age women with breast cancer in Croatia using a generic preference-based instrument (EQ-5D-5 L) and a breast cancer-specific module (EORTC QLQ-BR23). Despite their extensive international utilisation, no reference values exist for working-age women with breast cancer in Croatia.

Previous Croatian studies have assessed breast-cancer-related quality of life using EORTC QLQ-C30/BR23 in smaller, single-centre cohorts during adjuvant treatment and after mastectomy [10, 11], and reported health-state utilities using EQ-5D-3 L across localized and advanced disease stages [12]. However, to our knowledge, no multicentre study has reported EQ-5D-5 L utilities together with EORTC QLQ-BR23 profiles in a working-age Croatian cohort and benchmarked outcomes against national population norms.

## Methods

### Study design and participants

We conducted a multicentre, cross-sectional study including female patients with breast cancer treated at two large Croatian clinical centres—the University Hospital Centre Split and the University Hospital Centre Sestre milosrdnice in Zagreb—during 2024. Both institutions serve as national referral hubs and treat patients from across all Croatian regions, providing a geographically diverse cohort.

Eligible participants were women aged 18–65 years with a confirmed diagnosis of breast cancer who were managed in an outpatient ambulatory oncology setting at the participating centres, either (i) while receiving ongoing systemic anticancer treatment (including endocrine therapy where applicable) or (ii) during follow-up after completing active systemic anticancer treatment within the preceding 24 months. The 24-month criterion applied to women not receiving ongoing systemic treatment at the time of recruitment. Participants were approached during routine follow-up appointments or treatment-related outpatient visits. Eligibility was not restricted by disease stage (non-metastatic or metastatic) or tumour immunophenotype. Time since diagnosis refers to the year of the initial breast cancer diagnosis; therefore, long-term survivors could be included if they were still attending outpatient oncology care.

Women younger than 18 years, those who declined to provide written informed consent, patients who were not under active outpatient oncology care at the participating centres, and patients with severe comorbidities or documented psychiatric conditions that could compromise the validity of self-reported questionnaire data were excluded. This sampling approach ensured inclusion of working-age patients for whom quality of life assessment and survivorship support are clinically relevant, while maintaining a consistent outpatient care context across participants.

### Data collection procedures

Potential participants were first screened through hospital records at both study sites. Women who met the eligibility criteria were subsequently contacted by telephone and informed about the study procedures. Those who expressed willingness to participate were invited to complete a digital questionnaire including the EQ-5D-5 L and EORTC QLQ-BR23 during their next scheduled outpatient visit. At that time, written informed consent was obtained for both participation in the survey and extraction of relevant clinical information from hospital charts.

Each participant was assigned a study-specific identification code to ensure confidentiality. Clinical variables, including disease stage and treatment characteristics, were extracted from medical records by authorised medical personnel and entered into a secure database using this code. Patient-reported data, including all quality-of-life measures and sociodemographic variables, were collected independently through the electronic questionnaire on a tablet which did not ask for any personally identifying information. The linkage of clinical and survey datasets was performed exclusively via the unique identification codes. Additional sociodemographic questions regarding the perceived impact of breast cancer on marital status, household finances, and financial difficulty in making ends meet were self-developed for the purposes of this study. Sociodemographic variables, including age, education level, employment status, and financial situation, were collected as part of the patient-reported questionnaire.

## Measures

### Health-related quality of life—EQ-5D-5 L

The EuroQol 5-Dimension 5-Level questionnaire (EQ-5D-5 L) is a standardized and previously published generic health status instrument developed by the EuroQol Group [13]. The development and preliminary validation of the 5-level version have been described by Herdman et al. [13], and detailed guidance on administration and scoring is provided in the official EQ-5D-5 L User Guide [14]. The instrument is widely used in oncology research and clinical practice and enables comparability of HRQoL outcomes across studies and populations. It assesses health status across five dimensions—mobility, self-care, usual activities, pain/discomfort, and anxiety/depression—each with five response levels ranging from no problems to extreme problems. It also includes a visual analogue scale (VAS) ranging from 0 (worst imaginable health) to 100 (best imaginable health).

All obtained EQ-5D-5 L data were converted into utility index scores using the Slovenian EQ-5D-5 L value set developed by Prevolnik Rupel and Ogorevc [15], as a Croatian-specific value set is not currently available. Given the cross-sectional design of the study, HRQoL was measured at a single time point, and the resulting utility values were used to describe the sample’s health status and to explore associations with relevant sociodemographic and clinical variables.

### Breast cancer-related quality of life—EORTC QLQ-BR23

The European Organisation for Research and Treatment of Cancer Quality of Life Questionnaire–Breast Cancer Module (EORTC QLQ-BR23) is a previously published and validated 23-item disease-specific instrument developed by the EORTC Quality of Life Group [16]. It is widely used to evaluate HRQoL among women with breast cancer, focusing on breast cancer–specific domains. The questionnaire comprises functional scales (body image, sexual functioning, and future perspective) and symptom scales (systemic therapy side effects, breast and arm symptoms, and being upset by hair loss). Each item is scored on a 4-point Likert scale. Raw scores were linearly transformed to a 0–100 scale according to the EORTC scoring manual [17]. Higher scores on functional scales indicate better functioning and higher quality of life, whereas higher scores on symptom scales denote greater symptom severity or distress.

The EORTC QLQ-BR23 has been validated across different cultural contexts and populations [18] and complements the EQ-5D-5 L by capturing breast cancer–specific functional and symptom domains that are not fully reflected in generic HRQoL measures.

### Population norms

EQ-5D-5 L population reference values were obtained from a representative sample of Croatian women collected in 2022 (*n* = 471) using face-to-face interviews [19]. The reference dataset provides age-stratified norms for EQ-5D-5 L dimensions and EQ-VAS, enabling benchmarking of our working-age breast cancer cohort against women of comparable age in the general population. As before, the EQ-5D-5 L descriptive data were converted into utility index scores using the Slovenian EQ-5D-5 L value set developed by Prevolnik Rupel and Ogorevc [15].

### Statistical analysis

Age was categorised into quintiles based on the analytic sample distribution to create five groups of approximately equal size. Subgroup analyses were performed according to age groups and disease stage (metastatic vs. non-metastatic). Between-group comparisons used the Mann–Whitney U test (two groups) and the Kruskal–Wallis test (multiple groups). All tests were two-sided and statistical significance was set at *p* < 0.05. Analyses were performed using complete cases for each model (i.e., conducted on available data for each outcome - complete-case analysis and therefore denominators vary across tables depending on questionnaire completeness). No imputation was performed. The minimal important difference (MID) for EQ-5D-5 L utility in cancer populations has been estimated at 0.05–0.08; observed utility decrements exceeding this threshold are considered clinically meaningful [20, 21].

## Results

A total of 271 women participated in the study, having been recruited at the University Hospital Centre Split and University Hospital Centre Sestre milosrdnice, Zagreb, between January and December 2024. The baseline sociodemographic and clinical characteristics of the study population are presented in Table 1. The mean age of participants was 51; 45% had completed higher education; 21% reported some level of financial difficulty (Table 1). With respect to employment status, 71% of participants were employed. A minority of participants (8%) reported that the cancer diagnosis harmed their personal relationships, such as marital disruption or divorce. 45% reported that the diagnosis negatively affected their financial situation. The mean time since the first breast cancer diagnosis was 4.5 years (SD 3.57 years; median 2.5 years); 20% of participants had metastatic breast cancer (60% diagnosed with de novo metastatic cancer and 40% had experienced a relapse following an earlier diagnosis of localized disease). The most recent therapy was adjuvant treatment following surgery (76%); 87% had undergone surgical intervention, 45% had received chemotherapy and 59% had received adjuvant radiotherapy during the course of treatment.

**Table 1:** Basic sociodemographic and clinical characteristics of the study sample

Variable	Subcategory / Statistic	Value
Number of participants (N)		271
Age (years)	Range	24–65
	Mean	51.35
	Median	52
	Standard deviation	8.52
Highest level of education	Primary or less	9 (3.8%)
	Secondary school	121 (51.3%)
	Undergraduate degree	87 (36.9%)
	Postgraduate degree	19 (8.0%)
Has a breast cancer diagnosis negatively impacted your marital status?	No	216 (91.5%)
	Yes	20 (8.5%)
Has a breast cancer diagnosis negatively impacted household finances?	No	129 (54.7%)
	Yes	107 (45.3%)
How easy or difficult does your household make ends meet?	Extremely easy	3 (1.3%)
	Fairly easy	32 (13.6%)
	Neither difficult nor easy	140 (59.3%)
	Fairly difficult	46 (19.5%)
	Extremely difficult	4 (1.7%)
	Cannot decide	11 (4.7%)
Current employment status	Employed (including on sick leave)	165 (70.8%)
	Retired	29 (12.5%)
	Unemployed, seeking work	15 (6.4%)
	Housework/domestic work	19 (8.1%)
	Permanently unfit for work	5 (2.1%)
Duration since first breast cancer diagnosis (years)	Range	0–25
	Mean (SD)	4,5 (3.57)
	Median	2.5
Metastatic disease	No	219 (80.81%)
	Yes	52 (19.19%)
Systemic therapy in the past 24 months	Yes	265 (97.8%)
	No	6 (2.2%)
Surgery received	Yes	237 (87.5%)
	No	34 (12.5%)
Adjuvant radiotherapy	Yes	157 (57.9%)
	No	114 (42.1%)
Palliative radiotherapy	Yes	23 (8.6%)
	No	245 (91.4%)
Chemotherapy	Yes	121 (44.7%)
	No	150 (55.3%)
Most recent treatment line received	Adjuvant	207 (76.4%)
	Neoadjuvant	8 (3.0%)
	First line	35 (12.9%)
	Second line	14 (5.2%)
	Third line	4 (1.5%)
	Fourth or more lines	3 (1.1%)

Available-case denominators for self-reported sociodemographic items ranged from *n* = 233 to 236 because of item non-response; clinical variables were available for all 271 participants

### Breast cancer-related quality of life—EORTC QLQ-BR23

The EORTC QLQ-BR23 instrument (Table 2) indicated a moderate level of symptom burden and functional impairment across several HRQoL domains. Impairments were most pronounced for future perspective and sexual functioning, while symptom burden was notable for systemic therapy side effects and arm symptoms. Within the systemic therapy side-effects scale, a substantial proportion of participants reported experiencing some degree of treatment-related symptoms during the last week: dry mouth (63%), altered taste perception (41%), headaches (65%) and general malaise (69%). Vasomotor symptoms were also prevalent, with more than half of participants (58%) reporting hot flushes to some extent. Some degree of hair loss was noted by 48% of respondents and emotional distress related to alopecia was reported by nearly 40% of patients. Perceptions of body image were variably affected. Between 55% and 62% of women reported at least some degree of dissatisfaction with their appearance and diminished feelings of femininity during the last week. Sexual functioning scores, however, suggested considerable impairment. The majority of participants reported, during the previous 4 weeks, low levels of sexual interest (84%) and activity (84%), with a notable proportion (50%) of sexually active women describing reduced sexual enjoyment. Concerns regarding the future were also evident, with nearly two-thirds of participants expressing some level of worry about their health. Physical symptom burden related to the arm and breast was frequently reported. Approximately 70% of respondents experienced some degree of pain, swelling or movement limitation in the affected arm, and around 60% reported some degree of breast pain, swelling, oversensitivity, or skin changes.

**Table 2:** EORTC QLQ-BR23 instrument

Question No.	Results for each scale (Mean ± SD)	Question	Not at all (%)	A little (%)	Quite a bit (%)	Very much (%)
		During the past week:				
1	Systemic therapy side effects scale (32.33 ± 19.81)	Did you have a dry mouth?	37.29	33.9	23.73	5.08
2		Did food and drink taste different than usual?	59.32	21.61	14.83	4.24
3		Were your eyes painful, irritated or watery?	43.22	34.32	18.64	3.81
4		Have you lost any hair?	51.71	19.23	12.39	16.67
5	Concern about hair loss scale (38.55 ± 39.39)	Were you upset by the loss of your hair? (if applicable)	42.61	19.13	18.26	20.0
6	Systemic therapy side effects scale	Did you feel ill or unwell?	30.77	46.58	20.94	1.71
7		Did you have hot flushes?	15.81	26.07	38.89	19.23
8		Did you have headaches?	34.62	43.16	18.8	3.42
9	Body image scale (31.16 ± 29.8)	Have you felt physically less attractive as a result of your disease or treatment?	44.02	29.49	18.38	8.12
10		Have you been feeling less feminine as a result of your disease or treatment?	43.59	30.77	17.52	8.12
11		Did you find it difficult to look at yourself naked?	42.37	30.51	17.8	9.32
12		Have you been dissatisfied with your body?	38.46	35.9	16.67	8.97
13	Future perspective scale (40.63 ± 31.4)	Were you worried about your health in the future?	10.3	26.61	37.77	25.32
14	Sexual functioning and enjoyment scale (26.00 ± 24.15)	To what extent were you interested in sex? (past 4 weeks)	40.43	43.83	12.34	3.4
15		To what extent were you sexually active? (past 4 weeks)	40.95	43.1	13.79	2.16
16		Answer this question only if you have been sexually active: To what extent was sex enjoyable for you? (if applicable)	21.64	28.36	28.36	21.64
17	Arm symptom scale (33.24 ± 29.77)	Did you have any pain in your arm or shoulder?	29.49	29.49	27.35	13.68
18		Did you have a swollen arm or hand?	62.98	14.47	13.62	8.94
19		Was it difficult to raise your arm or move it sideways?	41.1	25.85	21.19	11.86
20	Breast symptom scale (26.16 ± 25.65)	Have you had any pain in the area of your affected breast?	36.75	41.45	15.38	6.41
21		Was the area of your affected breast swollen?	69.79	15.74	9.36	5.11
22		Was the area of your affected breast oversensitive?	40.85	30.21	17.02	11.91
23		Have you had skin problems in or around the area of your affected breast (e.g., itchy, dry, flaky)?	53.22	25.75	13.73	7.3

Scale-specific denominators were body image *n* = 227, future perspective *n* = 230, sexual functioning *n* = 229, sexual enjoyment *n* = 133, systemic therapy side effects *n* = 225, upset by hair loss *n* = 113, breast symptoms *n* = 227, and arm symptoms *n* = 230. Lower denominators for sexual enjoyment and upset by hair loss reflect item applicability and questionnaire completeness

Table 2 also reports compiled EORTC QLQ‑BR23 results across all eight scales/indices. Body image index confirms that patients report relatively low perceived attractiveness and satisfaction with physical appearance. Sexual functioning and enjoyment scale had a mean score of 26.0 (SD = 24.2) and 50.0 (SD = 35.3), respectively, indicating low sexual activity and enjoyment, consistent with known effects of breast cancer treatment on sexual health. The systemic therapy side effects scale had a mean score of 32.3 (SD = 19.8), indicating mild to moderate side effects associated with chemotherapy or other systemic therapies. The same applies to breast and arm symptoms, with a mean score of 26.2 (SD = 25.7) and 33.2 (SD = 29.8), respectively, indicating moderate arm and breast symptoms.

### Health-related quality of life—EQ-5D-5 L

**Fig. 1:**
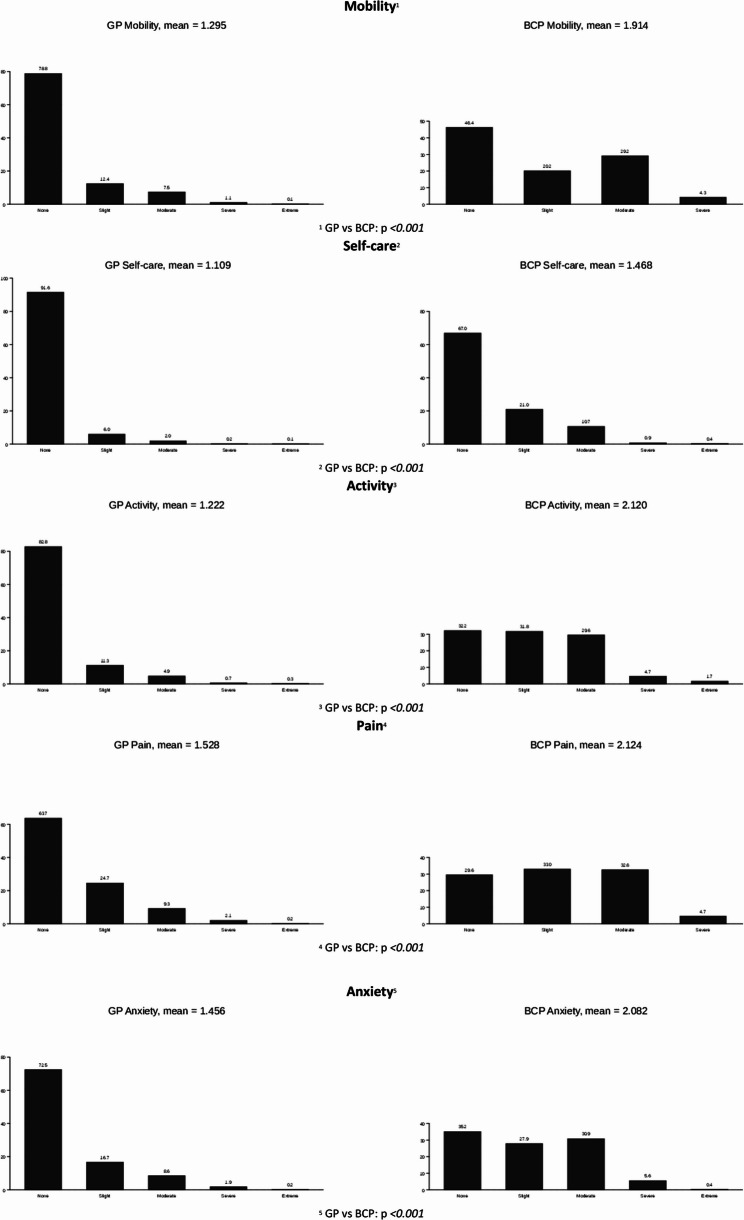
EQ-5D 5L dimensions breast cancer patients (BCP, n=233) and the general population (GP, 18-65, n=471)

Among the breast cancer patients (Fig. 1), the lowest level of impairment was observed in the self-care domain (mean = 1.47), where more than two-thirds of respondents reported no difficulties. In contrast, the pain/discomfort (mean = 2.12) and usual activities (mean = 2.12) dimensions showed the highest burden, with a visibly larger proportion of patients reporting moderate to severe problems (levels 3–5), seconded by the anxiety/depression (mean = 2.08) dimension.

Statistically significant differences were found across all EQ-5D-5 L dimensions and the visual analogue scale (VAS) between breast cancer patients and the general population (all *p* < 0.01, Table 3). Breast cancer patients reported higher problem levels across all EQ-5D-5 L dimensions than the general female population, including mobility (1.91 vs. 1.30), self-care (1.47 vs. 1.09), usual activities (2.12 vs. 1.22), pain/discomfort (2.12 vs. 1.53), and anxiety/depression (2.08 vs. 1.46) (Table 3; Fig. 1). These results are summarised in Table 3.

**Table 3:** EQ-5D-5 L outcomes in the general population (*n* = 471) and breast cancer cohort (*n* = 233)

Variable	General population					Breast cancer patients					
	*n*	Mean (SD)	Median (	Min	Max	*n*	Mean (SD)	Median	Min	Max	*p*-value
EQ-5D Index	471	0.91 (0.14)	1.00	-0.285	1.00	233	0.76 (0.19)	1.00	-0.01	1.00	< 0.01
VAS	471	83.99 (16.42)	90.00	10	100	233	66.17 (23.09)	66.0	0	100	< 0.01
Mobility	471	1.30 (0.64)	1.00	1	5	233	1.91 (0.96)	2.00	1	4	< 0.01
Self-care	471	1.09 (0.35)	1.00	1	5	233	1.47 (0.75)	1.00	1	5	< 0.01
Usual activities	471	1.22 (0.54)	1.00	1	5	233	2.12 (0.98)	2.00	1	5	< 0.01
Pain/Discomfort	471	1.53 (0.77)	1.00	1	5	233	2.12 (0.89)	2.00	1	4	< 0.01
Anxiety/Depression	471	1.46 (0.80)	1.00	1	5	233	2.08 (0.96)	2.00	1	5	< 0.01

**Fig. 2:**
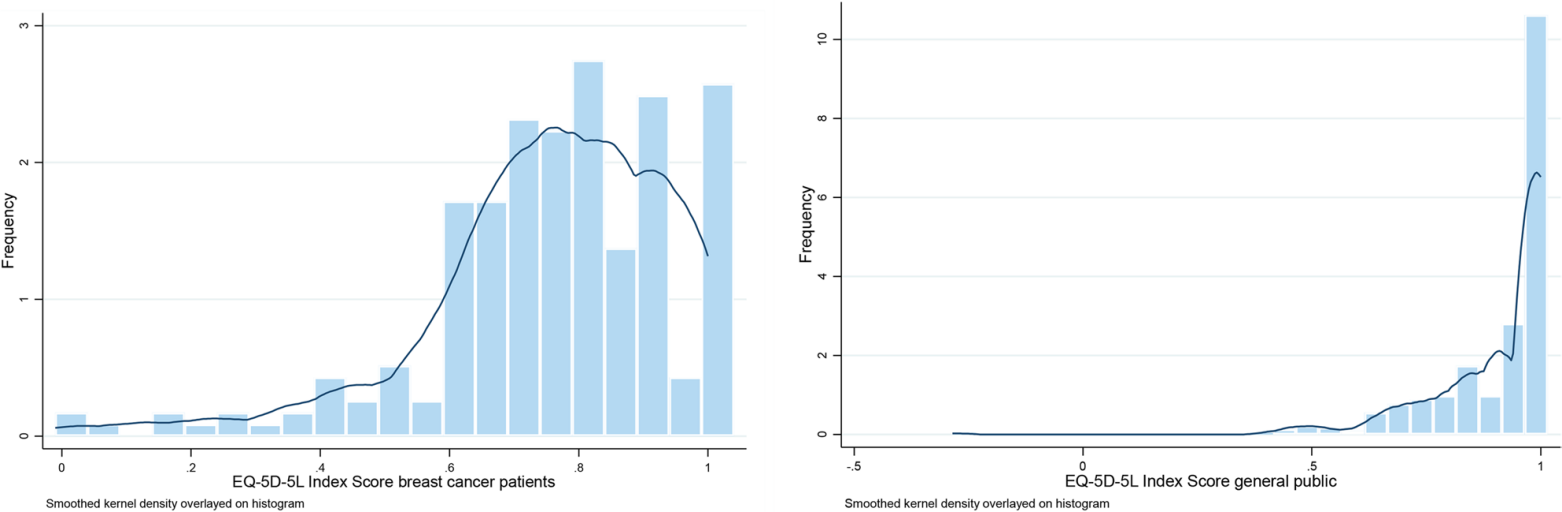
Distribution of EQ-5D-5L utility index in breast cancer patients (n=233) and the general population (n=471)

Correspondingly, the mean EQ-5D-5 L index among patients was 0.76, compared to 0.91 in the general female population of similar age, indicating a utility decrement of 0.15 on the EQ-5D-5 L index scale (Table 3). The EQ-VAS score was approximately 21% lower in breast cancer patients compared with the general female population (66.2 vs. 84.0), confirming substantially poorer self-rated health. Clinically, this magnitude of decrement exceeds the minimal important difference (MID) threshold of 0.05–0.08 typically cited for EQ-5D-5 L [20, 21], indicating a large and meaningful reduction in HRQoL. The distribution of the EQ-5D-5 L index (Fig. 2) reflects a subset of patients experiencing major decrements in health status.

### Health-related quality of life by age and disease type

Age-stratified analysis revealed a progressive decline in HRQoL with increasing age (Table 4). Mean EQ-5D-5 L index scores decreased from 0.82 in the youngest group (24–43 years) to 0.69 in the oldest (60–65 years) (*p* = 0.03). EQ-VAS scores followed a similar pattern, declining from 74 to 62 across age strata, though this trend did not reach significance (*p* = 0.08). Comparison by disease stage (Table 5) demonstrated a substantial decrement in HRQoL among patients with metastatic disease relative to those with non-metastatic disease. Mean EQ-5D-5 L and EQ-VAS scores were 0.70 and 0.58 for metastatic cases versus 0.77 and 0.68 for non-metastatic patients, respectively, corresponding to statistically significant differences (*p* < 0.05). Across the EORTC QLQ-BR23 domains, sexual functioning and sexual enjoyment (*p* = 0.01) declined significantly with advancing age, indicating age-related deterioration in sexual health and satisfaction. By contrast, no significant differences by age or metastatic status were detected for body image, future perspective, systemic-therapy side effects, concern regarding hair loss, breast symptoms, or arm symptoms (all *p* > 0.05).

**Table 4:** HRQoL domains across age-based subgroups

Groups	Age 24–43				Age 44–48				Age 49–54				Age 55–59				Age 60–65					
Variable	*n*	Mean (sd)	Min	Max	*n*	Mean (sd)	Min	Max	*n*	Mean (sd)	Min	Max	*n*	Mean (sd)	Min	Max	*n*	Mean (sd)	Min	Max	KW χ²	*p*
EQ-5D index	35	0.82 (0.15)	0.36	1.00	53	0.79 (0.15)	0.28	1.00	56	0.78 (0.17)	0.09	1.00	41	0.71 (0.22)	0.04	1.00	48	0.70 (0.23)	−0.01	1.00	10.38	0.03
VAS	35	72.00 (25.08)	0	100	53	65.51 (24.40)	6	100	56	69.91 (19.41)	5	100	41	61.17 (25.50)	2	99	48	62.54 (21.10)	5	100	8.352	0.08
EQ-5D index general population	248	0.95 (0.10)	0.41	1.00	71	0.91 (0.11)	0.48	1.00	45	0.86 (0.16)	0.49	1.00	56	0.84 (0.16)	0.43	1.00	51	0.82 (0.21)	−0.29	1.00	99.08	0.00
VAS general population	248	89.06 (13.61)	45	100	71	83.61 (16.51)	25	100	45	78.62 (16.88)	50	100	56	76.73 (15.81)	20	100	51	72.55 (19.11)	10	100	141.83	0.00
Body image scale	34	30.39 (29.86)	0	100	51	37.58 (33.06)	0	100	55	32.27 (26.98)	0	100	41	26.63 (29.53)	0	100	46	29.17 (29.59)	0	100	3.68	0.45
Future perspective scale	35	40.00 (30.03)	0	100	53	39.62 (34.00)	0	100	56	40.48 (30.95)	0	100	40	40.00 (28.44)	0	100	46	41.30 (31.57)	0	100	0.17	0.99
Sexual functioning scale	35	34.29 (27.10)	0	100	52	30.13 (24.49)	0	100	55	26.36 (23.50)	0	100	40	25.00 (20.67)	0	66.7	47	17.38 (22.78)	0	100	12.40	0.01
Sexual enjoyment scale	22	63.64 (32.38)	0	100	32	57.29 (34.11)	0	100	32	48.96 (31.66)	0	100	25	52.00 (37.37)	0	100	22	27.27 (33.55)	0	100	13.48	0.01
Systemic therapy side effects scale	34	29.13 (18.15)	0	71.4	51	26.89 (17.66)	0	80.95	53	36.57 (20.96)	0	85.71	41	36.59 (21.89)	0	85.71	46	31.16 (17.89)	0	71.43	8.28	0.08
Concern by hair loss scale	16	47.92 (40.31)	0	100	19	38.60 (40.47)	0	100	26	35.90 (38.78)	0	100	24	33.33 (39.32)	0	100	28	41.67 (41.20)	0	100	1.50	0.83
Breast symptoms scale	35	21.90 (18.53)	0	66.7	52	26.12 (24.37)	0	100	55	30.15 (30.41)	0	100	40	28.13 (28.79)	0	100	45	23.89 (23.27)	0	91.7	0.83	0.94
Arm symptom scale	35	30.16 (27.56)	0	100	53	31.45 (32.81)	0	100	56	35.32 (32.56)	0	100	39	39.32 (29.60)	0	100	47	30.50 (25.32)	0	88.9	2.72	0.61

KW -Kruskal–Wallis equality-of-populations rank test χ² - chi2

**Table 5:** HRQoL domains across disease types

Variable	Non-metastatic disease					Metastatic disease				
	*n*	Mean (SD)	Min	Max	*n*	Mean (SD)	Min	Max	KW χ² (df = 1)	*p*-value
EQ-5D index	190	0.77 (0.18)	0.09	1.00	43	0.70 (0.21)	−0.01	1.00	4.93	0.03
VAS	190	67.96 (22.52)	0	100	43	58.23 (24.16)	2	95	6.69	0.01
Body image	185	31.35 (29.80)	0	100	42	32.34 (30.20)	0	100	0.02	0.90
Future perspective	187	41.18 (30.69)	0	100	43	36.43 (32.38)	0	100	1.13	0.29
Sexual functioning	186	27.69 (24.37)	0	100	43	20.54 (22.37)	0	83.3	3.27	0.07
Sexual enjoyment	108	51.54 (34.52)	0	100	25	45.33 (38.35)	0	100	0.58	0.45
Systemic therapy side effects	183	31.88 (19.19)	0	85.7	42	33.33 (21.76)	0	85.7	0.04	0.85
Upset by hair loss	86	37.98 (39.66)	0	100	27	41.98 (39.86)	0	100	0.26	0.61
Breast symptoms	186	26.79 (25.92)	0	100	41	24.39 (25.23)	0	100	0.48	0.49
Arm symptoms	187	34.28 (30.14)	0	100	43	29.20 (29.10)	0	100	1.03	0.31

## Discussion

The results of this study reveal a substantial decrement in health-related quality of life among Croatian women with breast cancer, with a mean EQ-5D-5 L utility score of 0.76 compared with 0.91 in the general female population, representing a clinically meaningful reduction in HRQoL. Pain/discomfort, usual activities, and anxiety/depression were the most affected EQ-5D-5 L domains; more than half of respondents reported at least slight problems in these dimensions, and roughly one third reported moderate-to-extreme problems.

These findings align with previous European and international studies demonstrating persistent physical, emotional, and functional burdens long after completion of primary therapy [5, 22]. The magnitude of the decrement observed in our study exceeds commonly cited minimal important difference thresholds for EQ-5D-5 L (0.05–0.08) [20, 21], underscoring its clinical relevance. If sustained over one year, this utility decrement conceptually corresponds to approximately 0.15 quality-adjusted life-years (QALYs) (≈ 55 quality-adjusted days), noting this is an illustrative conversion rather than a directly measured annual loss.

Beyond the generic utility decrement captured by the EQ-5D-5 L, disease-specific assessment using the EORTC QLQ-BR23 revealed additional dimensions of survivorship burden that are not fully reflected in generic HRQoL measures. In particular, impairments were observed across domains related to physical symptoms in the arm and breast, body image and sexual functioning, highlighting the multidimensional impact of breast cancer and its treatment. These findings underscore the importance of complementing generic utility instruments with disease-specific tools when evaluating quality of life in breast cancer survivors, as reliance on generic measures alone may underestimate clinically relevant functional and psychosocial deficits that persist beyond primary treatment [16, 18].

Sexual health emerged as one of the most affected domains (mean QLQ-BR23 sexual functioning score 26/100), consistent with previous evidence showing that body image disturbances, reduced sexual interest and treatment-related menopausal symptoms are common and may persist after breast cancer diagnosis and treatment [23, 24]. This is also consistent with large Scandinavian survivorship data showing high prevalence of sexual inactivity and persistent sexual health concerns many years after diagnosis using EORTC QLQ-BR23 metrics [25]. Given that 84% of participants reported low sexual interest and activity, this reinforces the need for routine incorporation of sexual health counselling, body-image rehabilitation and psychosexual therapy within survivorship programmes.

The domain pattern observed in EQ-5D-5 L (pain/discomfort and anxiety/depression showing greatest impairment) is clinically plausible and may be driven by recognised long-term sequelae such as postmastectomy pain syndrome [26], chemotherapy-induced peripheral neuropathy and its impact on daily functioning and quality of life [27], aromatase inhibitor-associated arthralgia [28], and fear of cancer recurrence, which is linked to reduced quality of life in long-term breast cancer survivors [29]. Taken together, these findings support a survivorship model that systematically identifies symptoms and distress and routes patients into targeted supportive services—an approach consistent with expert survivorship consensus statements emphasising physical and psychological late effects as core components of survivorship care [30].

Age-stratified analyses demonstrated a progressive decline in HRQoL with increasing age, yet even the youngest women showed substantial impairments relative to general population norms (EQ-5D index 0.82 at age 24–43 years). This is consistent with prior evidence that age and treatment-related factors are important correlates of HRQoL in women with breast cancer, indicating that survivorship burden reflects disease- and treatment-related factors in addition to demographic influences [22]. The observed pattern of age-related decline in sexual functioning (Kruskal-Wallis *p* = 0.01) may reflect cumulative treatment effects, natural menopausal status, and evolving body image concerns over time. Although older women demonstrated lower absolute HRQoL scores, the relative decrement compared with age-matched general population norms was comparable across age groups. This indicates that the survivorship-related quality-of-life burden is substantial across the working-age spectrum rather than being driven predominantly by age. Financial difficulties reported by a proportion of participants may represent an additional source of psychosocial burden and could further negatively affect HRQoL. In addition, our findings suggest that the decline in HRQoL associated with aging may be further compounded by the burden of breast cancer, as health utility values remained consistently below population norms across all age groups.

Metastatic disease status was significantly associated with poorer HRQoL outcomes (EQ-5D index 0.70 vs. 0.77; *p* = 0.03), consistent with expectations that advanced disease carries greater functional and psychological burden [31]. While not reaching statistical significance for all QLQ-BR23 domains (likely due to the smaller metastatic subgroup, *n* = 43), trends toward greater impairment in sexual functioning and lower future perspectives in metastatic patients align with evidence of higher symptom burden and end-of-life concerns in advanced breast cancer [32].

These results provide a useful regional reference point for HRQoL research in breast cancer survivorship, facilitating cross-country comparisons, particularly within Central and Eastern Europe. Policy integration of routine patient-reported outcomes (PROs) monitoring in oncology practice represents an important next step for translating evidence into sustainable improvements in survivorship care quality. In practical terms, this also supports implementing structured distress screening and management pathways as part of routine PROs monitoring, consistent with established distress-management recommendations [33, 34].

Routine PRO monitoring is increasingly supported by European guidance and policy initiatives, including the European Society for Medical Oncology (ESMO) Clinical Practice Guideline on patient-reported outcome measures (PROMs) and European Cancer Organisation policy recommendations that emphasize workflow redesign and trained staff (e.g., nurses and allied health professionals) to review and act on PRO alerts [35, 36].

Evidence from Europe also demonstrates that electronic patient-reported outcomes (ePRO) implementation can be feasible and clinically beneficial: the phase III eRAPID trial (UK) improved physical well-being early during chemotherapy and increased patient self-efficacy without increasing hospital workload, while the European multicentre eSMART trial (Advanced Symptom Management System; ASyMS) reduced symptom burden and improved HRQoL and anxiety outcomes during chemotherapy [37, 38].

In addition to trial evidence, several European health systems have implemented PRO-driven care models at scale, including the Danish AmbuFlex telePRO approach for PRO-based follow-up and large routine-care remote symptom monitoring pathways such as a nurse navigator-supported weekly ePRO programme implemented across 33 cancer centres in France and Belgium, with reimbursement approved in France [39, 40].

Finally, a recent multinational EORTC randomised trial showed that providing clinicians access to patients’ symptom PRO data improves the consistency of Common Terminology Criteria for Adverse Events (CTCAE) toxicity grading for the majority of symptomatic adverse events, reinforcing the value of systematically incorporating the patient perspective into routine clinical evaluation [41].

From a clinical survivorship perspective, our pattern of impairment (particularly pain/discomfort, anxiety/depression, and marked sexual functioning deficits) supports structured aftercare programmes that combine routine PRO screening with predefined referral pathways including psychosocial care, symptom management/rehabilitation, and sexual health support [30, 35, 42].

Such models are consistent with emerging nurse-led survivorship follow-up strategies that use PROs as a dialogue and triage tool. For example, the MyHealth phase III trial demonstrated that a nurse-led programme incorporating self-management sessions, regular symptom reporting, and navigation to services improved breast cancer-specific quality of life and reduced fear of recurrence, anxiety and depression compared with physician-led follow-up [42].

Earlier European follow-up trials also indicate that some scheduled clinic visits can be safely replaced by nurse-led telephone follow-up without deterioration in HRQoL, supporting resource-efficient follow-up models when coupled with systematic symptom assessment and clear escalation criteria [43].

Further studies in Croatia and comparable Central and Eastern European settings should include longitudinal HRQoL follow-up to define symptom trajectories and high-risk subgroups, and pragmatic trials evaluating whether PRO-triggered survivorship pathways improve patient-important outcomes and reduce avoidable acute care use. In addition, hybrid effectiveness–implementation studies are needed to evaluate adoption, reach, equity, cost-effectiveness, and sustainability of digital PRO systems within real-world oncology workflows [35, 36, 40].

### Study strengths and limitations

This study’s key strengths include a relatively large, multicentre sample (*n* = 271) drawn from two major oncology centres, capturing patients from a broad geographic area across Croatia. The simultaneous assessment of health-related quality of life using both a generic and a breast cancer-specific instrument enables a comprehensive evaluation of the societal burden of breast cancer beyond direct clinical outcomes. The use of validated, internationally recognised instruments (EQ-5D-5 L, EORTC QLQ-BR23), together with comparison against population-based EQ-5D-5 L norms from a nationally representative 2022 survey, strengthens the interpretation and policy relevance of the estimated utility losses and functional deficits.

Several limitations should be considered. First, the cross-sectional design precludes causal inference and does not capture changes in HRQoL over time following diagnosis or treatment completion. This means that observed associations—such as lower HRQoL in older patients or those with metastatic disease—should be interpreted as differences between groups at a single time point rather than evidence of causal relationships, and may be influenced by unmeasured confounding factors including treatment history, comorbidities, and socioeconomic status. Second, this study utilises the Slovenian EQ-5D-5 L value set rather than a Croatian-specific tariff, which does not exist. While the Slovenian set is reasonable for CEE populations, values may differ from those derived from a Croatian contingent valuation study. In addition, population norms were collected via face-to-face interviews, whereas patient-reported outcomes were collected electronically during outpatient visits; potential mode effects in self-reporting cannot be excluded. Third, the study population consisted of women attending two tertiary oncology centres, which may not be fully representative of all Croatian breast cancer survivors, particularly those in primary care or private settings. Fourth, subgroup comparisons were unadjusted and residual confounding (e.g., age, socioeconomic status, comorbidities, and treatment-related factors) cannot be excluded. Additionally, multiple HRQoL domains were tested without formal multiplicity adjustment; findings should therefore be interpreted as hypothesis-generating. We used the EORTC QLQ-BR23 module. Although an updated breast cancer module (EORTC QLQ-BR42) is now available [44], QLQ-BR23 remains widely used and validated, allowing comparability with prior literature [16–18]. We did not administer the core EORTC QLQ-C30 questionnaire to minimise respondent burden; therefore, global QoL and some general cancer-related domains were not assessed.

Despite these limitations, this study provides multicentre benchmark HRQoL data for working-age Croatian breast cancer survivors, including preference-based utility estimates contextualised against Croatian population norms and detailed breast cancer–specific domains.

## Conclusions

This study provides baseline health-related quality of life reference values for working-age women with breast cancer in Croatia, demonstrating a clinically meaningful reduction in utility compared with population norms. Persistent impairments across physical, emotional and disease-specific domains highlight substantial unmet survivorship care needs beyond completion of primary treatment. Integration of routine patient-reported outcome monitoring into oncology follow-up, alongside structured psychosocial support, sexual health counselling and rehabilitation services, may help address these long-term challenges. Based on the observed gradients in HRQoL, survivorship support services should prioritise women with metastatic disease, as well as age groups showing the largest absolute deviations from population norms, in order to address the highest unmet supportive care needs. Specifically, implementation of routine distress screening and evidence-based management of anxiety/depression [33, 34], together with guideline-concordant survivorship follow-up addressing late effects, sexual health, and functional problems [45], could directly target the most affected domains observed in this cohort.

## Data Availability

The datasets generated and/or analysed during the current study are not publicly available due to ethical restrictions and the sensitive nature of the data but are available from the corresponding author on reasonable request, subject to approval by the relevant ethics committee.

## Abbreviations

ASyMS: Advanced Symptom Management System
CEE: Central and Eastern Europe
CTCAE: Common Terminology Criteria for Adverse Events
df: Degrees of freedom
ePRO: Electronic patient–reported outcomes
EORTC: European Organisation for Research and Treatment of Cancer
EQ: 5D–3 L–EuroQol 5–Dimension 3–Level
EQ: 5D–5 L–EuroQol 5–Dimension 5–Level
EQ: VAS–EuroQol Visual Analogue Scale
ESMO: European Society for Medical Oncology
HRQoL: Health–related quality of life
KW: Kruskal–Wallis (test)
MID: Minimal important difference
PRO(s): Patient–reported outcome(s)
QALY: Quality–adjusted life year
QLQ: BR23–EORTC Quality of Life Questionnaire–Breast Cancer Module (BR23)
QLQ: BR42–EORTC Quality of Life Questionnaire–Breast Cancer Module (BR42; updated module)
QLQ: C30–EORTC Quality of Life Questionnaire Core 30
SD: Standard deviation
VAS: Visual analogue scale
χ²: Chi–square
